# Large Language Models in Preclinical Spine Research: A Scoping Review and Expert Perspective on Evidence‐Aware Experimental Workflows

**DOI:** 10.1002/jsp2.70203

**Published:** 2026-07-08

**Authors:** Siegmund Lang, Stefan Motov, Jonas Krueckel, Julian Scherer, Victor E. Staartjes, Karin Wuertz‐Kozak

**Affiliations:** ^1^ Department of Trauma Surgery University Hospital Regensburg Regensburg Germany; ^2^ Spine Center of Eastern Switzerland & Department of Neurosurgery Kantonsspital St. Gallen, Medical School of St. Gallen, University of St. Gallen St. Gallen Switzerland; ^3^ Department of Traumatology University Hospital Zurich, University of Zurich Zurich Switzerland; ^4^ Center for Musculoskeletal Surgery, Charité – Universitätsmedizin Berlin Berlin Germany; ^5^ Department of Surgery Spital Uster Uster Switzerland; ^6^ Machine Intelligence in Clinical Neuroscience (MICN) & Microsurgical Neuroanatomy Laboratory, Department of Neurosurgery Clinical Neuroscience Center, University Hospital Zurich, University of Zurich Zurich Switzerland; ^7^ Department of Clinical Neuroscience Karolinska Institutet Stockholm Sweden; ^8^ Department of Biomedical Engineering Rochester Institute of Technology Rochester New York USA

## Abstract

**Background:**

Preclinical spine research is limited by heterogeneous experimental reporting, fragmented documentation, and barriers to reproducibility and translational alignment. Large language models (LLMs) and related artificial intelligence (AI) technologies may support semantic interpretation, structured data extraction, and reasoning over biomedical text, but their role in experimental spine science remains unclear. This focused scoping review and expert perspective mapped current AI/LLM applications in spine research, quantified the preclinical evidence gap, and identified responsible integration opportunities.

**Methods:**

A structured search of PubMed, Embase, and Web of Science was performed for studies published from January 2020 to January 2026 evaluating LLM, AI, chatbot, or advanced natural language processing applications in spine‐related contexts. The search intentionally captured both LLM‐specific and broader AI/chatbot applications to map the translational landscape. For this preclinical‐focused analysis, the corpus was re‐examined for experimental and translational use cases, supplemented by expert synthesis of methodologically relevant adjacent biomedical literature.

**Results:**

Of 792 records identified, 166 unique studies met inclusion criteria. Publication activity increased markedly over time. The literature was dominated by conversational assessment/patient‐reported outcome measure applications (82/166; 49.4%), patient education/information quality studies (53/166; 31.9%), and other LLM/AI applications (19/166; 11.4%). Preclinical/basic science applications were rare (3/166; 1.8%) and used classical machine learning, deep learning, or broader AI frameworks rather than generative LLMs. The most credible near‐term opportunities include schema‐constrained data extraction, protocol completeness checking, ontology‐aligned data structuring, and evidence‐grounded workflow support under human supervision.

**Conclusion:**

In preclinical spine research, LLMs are best positioned as human‐supervised workflow instruments for structuring fragmented experimental knowledge. Spine‐specific validation and robust governance are essential for responsible translational use.

## 1 Introduction

Spine research is characterized by rapidly expanding data volumes, increasing subspecialization, and persistent heterogeneity in terminology, outcomes, and reporting standards. Nevertheless, we face an evident translational problem that is often framed as a biological one: Animal models are imperfect, disease mechanisms are multifactorial, and clinical outcomes are noisy. Traditional evidence synthesis, based on keyword searches, manual screening, and conventional review methods, remains robust for narrowly framed questions but is poorly suited to integrate semantically related evidence across heterogeneous vocabularies and clinical contexts. This limitation is particularly relevant in spine science, where core constructs such as instability, sagittal balance, and fusion success are operationalized inconsistently across trauma, deformity, degenerative, infectious, and oncologic domains, thereby impeding cumulative knowledge generation. Much of what we call “phenotype” in spine care is not a direct or singular measurement but a layered and quite involved description assembled from symptoms, physical examination, and imaging/intraoperative impressions. Preclinical work, in turn, describes constructs that are partly overlapping but expressed in a different language, comprising histology, behavior, induced injury paradigms, and mechanistic readouts. Large language models (LLMs) and the broader family of advanced AI systems built on similar architectural principles introduce a fundamentally novel paradigm by encoding biomedical text as contextual semantic embedding rather than isolated keywords. Transformer‐based systems enable concept‐aware retrieval and cross‐terminology reasoning, and sufficiently scaled instruction‐tuned models have demonstrated the capacity to encode clinically relevant knowledge patterns across medical domains [1, 2]. At the same time, recent studies suggest that even advanced medical LLMs remain inferior to clinicians in complex reasoning tasks and therefore require rigorous human oversight and domain alignment before clinical deployment [3].

Beyond retrieval and structured extraction, modern transformer‐based LLMs increasingly exhibit emergent reasoning capabilities relevant to preclinical research workflows. Instruction‐tuned and sufficiently scaled models demonstrate chain‐of‐thought prompting and multi‐step logical inference that enable evaluation of experimental designs against established criteria, identification of logical gaps in protocols, and structured comparison of competing mechanistic hypotheses. Agentic LLM architectures further extend this capacity by invoking external tools, for example, calling retrieval engines, code‐execution environments, and structured database queries, within an iterative reasoning loop. Importantly, these capabilities remain imperfect and calibration‐limited rather than absent. Failure modes such as overconfident but unsupported claims, numerical confabulation, and citation misattribution have been characterized and can be mitigated through retrieval‐augmented generation, predefined human verification checkpoints, and provenance tagging.

LLMs have been proposed as semantic interfaces capable of mapping synonymous concepts, detecting latent relationships, and generating literature‐derived hypothesis spaces. They offer a new class of computational methods that can act on meaning‐bearing text on a scale. In other words, they operate on the “translation layer” that sits between measurements and conclusions/interpretations.

### 1.1 Objective of This Scoping Review

The objective of this scoping review is to delineate and critically contextualize the emerging roles of LLMs within preclinical and experimental spine research. Specifically, we aim to map high yield use cases across the preclinical research lifecycle, evaluate current evidence maturity, and identify methodological constraints that may influence reliability and reproducibility. By situating LLMs within the broader challenges of experimental heterogeneity, semantic fragmentation, and data interoperability, this review seeks to provide a pragmatic framework for responsible, human‐supervised integration of LLMs into preclinical spine science and translational research workflows.

## 2 Methods

This manuscript was conducted as a focused scoping review complemented by an expert perspective. The original scoping framework was structured around two overarching domains: (A) preclinical spine research and (B) clinical spine care and research. The clinical domain (Part B) will be reported separately in a dedicated companion manuscript. The rationale for this separation is that preclinical and clinical domains raise fundamentally different methodological questions—reproducibility, Findable–Accessible–Interoperable–Reusable (FAIR) data alignment, Replacement–Reduction–Refinement (3Rs) optimization, and protocol standardization versus patient education, clinical decision support, and imaging—and target distinct readerships. The structured search strategy and study selection process were intentionally designed to capture the full translational landscape of LLM and AI applications in spine research and care. The search included both LLM‐specific terms and broader AI/chatbot terminology to ensure comprehensive landscape mapping, as these terms are often used interchangeably in the primary spine literature. For the purposes of the present preclinical analysis, the mapped corpus was re‐examined with specific emphasis on preclinical and experimental use cases. Studies that were methodologically most informative or representative of a given thematic category were selected for citation in the expert synthesis sections, while the included studies were systematically characterized in the data‐charting table (Data S3). The structured search served two functions: [1] quantifying the evidence landscape and identifying the gap in preclinical applications, and [2] informing and complementing the expert‐driven thematic synthesis.

### 2.1 Structured Literature Search Strategy

To provide an empirical foundation for the narrative synthesis, a structured literature search was performed in PubMed (MEDLINE), Embase (Ovid), and Web of Science Core Collection. The final search was conducted on January 15, 2026 and covered publications from January 2020 to January 2026, reflecting the emergence of modern large language models. Searches were limited to English and German language publications. The search strategy combined free‐text terms related to large language models and conversational AI (including “large language model,” LLM, ChatGPT, GPT‐4, Claude, Gemini, Llama, Med‐PaLM, chatbot, and conversational AI) with spine‐specific terminology and terms reflecting either clinical or experimental contexts (Data S1). To ensure specificity and avoid retrieval of unrelated artificial intelligence literature, searches were restricted to title and abstract fields in PubMed and Embase and to topic fields in Web of Science. Broad artificial intelligence indexing terms were intentionally excluded.

All identified records were imported into Covidence for centralized management and automatic deduplication. Titles and abstracts were screened independently by two reviewers, followed by independent duplicate full‐text assessment by two reviews. Disagreements were resolved by consensus. Studies were considered eligible if they reported development, evaluation, benchmarking, or implementation of language‐model–based systems in spine surgery, spine patient care, or spine‐related preclinical and translational research contexts. Both clinical and laboratory‐based applications were included. Studies were excluded if they were non‐English or non‐German publications, purely imaging‐based AI investigations without language‐model components, robotics‐only studies, or opinion pieces without methodological content.

### 2.2 Reporting Framework

This scoping review was reported in accordance with the Preferred Reporting Items for Systematic Reviews and Meta‐Analyses extension for Scoping Reviews (PRISMA‐ScR) [4]. A completed PRISMA‐ScR checklist is provided as Data S2. Because the review was designed as a focused scoping review complemented by expert perspective, formal critical appraisal of individual sources of evidence was not performed; this is consistent with the exploratory and mapping‐oriented purpose of the review. Items not applicable to the present hybrid design are indicated in the checklist with justification. No formal review protocol was registered for this focused scoping review and expert perspective.

### 2.3 Data Charting and Thematic Categorization

The included studies were charted by two reviewers independently into the following data fields: First author, year, journal, AI/LLM system(s) used, AI application characterization (generative LLM, unspecified LLM, conventional chatbot/non‐LLM, classical/other AI/ML, hybrid LLM + ML/DL, AI general/unclear, or not specified/unclear), thematic domain, study design, and spine pathology focus (Data S3). Thematic domains were defined a priori and refined iteratively during charting, resulting in the following categories: [1] conversational assessment/PROM applications; [2] patient education/information quality; [3] other LLM/AI applications; [4] preclinical/basic science; [5] imaging‐focused AI/ML; [6] predictive modeling/decision support; [7] surgical documentation/planning; [8] patient Q&A/clinical vignette evaluation; and [9] informed consent/shared decision‐making. Discrepancies in categorization were resolved by consensus. Where the AI system could be identified from the title and abstract (e.g., GPT‐3.5, GPT‐4, Gemini, Claude, DeepSeek), this was recorded. Studies were further classified as using LLM‐based systems, classical ML/DL systems, or conventional (non‐LLM) chatbots to enable stratification as recommended by the reviewers. This distinction was not always possible from the primary literature, as many spine studies use the terms “chatbot,” “conversational AI,” and “LLM” interchangeably without specifying the underlying architecture. Charted data were summarized descriptively using frequencies and percentages for major application domains. Preclinical/basic science studies were then examined narratively in greater detail, and representative adjacent‐domain studies were used to inform the expert‐perspective synthesis.

#### 2.3.1 Expert‐Led Narrative Synthesis

Given that only three studies directly addressed preclinical spine applications (Table 1), the expert‐driven thematic synthesis was conducted by the author team to identify transferable principles and actionable frameworks from adjacent biomedical domains. The author team comprises researchers with complementary expertise spanning preclinical spine research (intervertebral disc biology, spinal fusion, spinal cord injury), clinical spine surgery (trauma surgery, neurosurgery, deformity surgery), AI/ML methodology and clinical neuroscience, and biomedical engineering. Themes for the expert synthesis were selected based on the intersection of identified evidence gaps from the scoping search and the stages of the preclinical research lifecycle (evidence synthesis, study design, protocol formalization, experimental execution, data integration, and reporting). The expert synthesis sections are structurally separated from the scoping review results and labeled as “Expert Perspective”. These sections draw on both the included corpus and methodologically relevant literature from adjacent fields, with citations to the broader biomedical AI literature used to illustrate transferable principles rather than spine‐specific empirical evidence.

**TABLE 1:** Characteristics and key findings of the three preclinical/basic science studies identified in the scoping review.

Study	Study type	AI/ML system	Preclinical application	Key findings for preclinical use	Limitations
Melgoza et al. (2021) JOR Spine [5]	Original research; ML algorithm development and validation	Supervised/unsupervised ML; artificial neural networks	Standardized histopathology scoring for murine IVD degeneration	High sensitivity/specificity for quantitating degenerative changes; reduces inter‐observer variability; enables standardized, automated phenotyping in preclinical disc models	Single‐species (mouse); limited to histopathology; no LLM component; requires validation across other species/models
Alini et al. (2023) JOR Spine [6]	Narrative review with expert perspective	AI, deep learning, convolutional neural networks, facial recognition algorithms	AI‐assisted histopathology scoring (murine, ovine); facial recognition pain assessment; DL‐enhanced spinal imaging	AI improves sensitivity/accuracy of histological and imaging evaluation; facial recognition enables objective pain assessment; AI represents paradigm shift for resolving IVD degeneration complexity	Narrative review without systematic search; AI applications discussed prospectively; no LLM‐specific evaluation; facial recognition may be more suitable for acute than chronic pain
Maity et al. (2026) J Clin Med [2]	Perspective article with structured literature search	DL, CNN, NLP, LLMs (ChatGPT, DeepSeek), unsupervised clustering	Hybrid modeling; NLP‐based data extraction; LLM‐driven structured text analysis; unsupervised phenotyping	AI matured from proof‐of‐concept to expert‐level performance in imaging; NLP enables automated registry construction from unstructured text; LLMs show promise for structured data extraction; hybrid models improve multimodal integration	Primarily clinically focused; preclinical applications extrapolated rather than directly evaluated; generalizability and external validation remain major barriers; LLM accuracy for patient education still insufficient

Abbreviations: AI, artificial intelligence; CNN, convolutional neural network; DL, deep learning; IVD, intervertebral disc; LLM, large language model; ML, machine learning; NLP, natural language processing.

### 2.4 Abbreviations

All abbreviations and technical terms used throughout the manuscript are summarized in the glossary (Table 2).

**TABLE 2:** Glossary.

Abbreviation	Spelled‐out meaning	Brief explanation
μCT	Micro‐computed tomography	High‐resolution computed tomography used for small specimens or preclinical models
3Rs	Replacement, reduction, and refinement	Ethical framework guiding animal research
AE	Adverse event	Undesirable event occurring during or after an intervention or experimental procedure
AI	Artificial intelligence	Umbrella term for computer systems performing tasks that typically require human intelligence
AlphaFold	AlphaFold (DeepMind protein structure prediction model)	Deep learning model predicting protein 3D structures with high accuracy
ARRIVE	Animal research: reporting of in vivo experiments	Reporting guideline for animal experiments
BERT	Bidirectional encoder representations from transformers	Transformer model enabling bidirectional contextual language understanding
BioASQ	Biomedical semantic indexing and question answering	Benchmark challenge for biomedical semantic indexing and QA
BioGPT	Biomedical generative pre‐trained transformer	Domain‐specific generative transformer for biomedical literature mining
Chain‐of‐thought	Chain‐of‐thought reasoning	Prompting strategy enabling multi‐step reasoning in LLMs
ChatGPT	Chat generative pre‐trained transformer	Conversational AI system based on GPT large language models
CLIP	Contrastive language–image pretraining	Vision‐language model aligning image and text representations
CNN	Convolutional neural network	Deep learning architecture commonly used for image analysis
CONSORT‐AI	Consolidated standards of reporting trials–artificial intelligence	Reporting extension for clinical trials involving AI interventions
Coscientist	Coscientist	GPT‐driven autonomous research support system for experimental design
DL	Deep learning	Multi‐layer neural network–based machine learning approach
DOI	Digital object identifier	Persistent identifier for scholarly publications
EQUATOR	Enhancing the QUAlity and transparency of health research	International network and repository for reporting guidelines
FAIR	Findable–accessible–interoperable–reusable	Data stewardship principles enabling reusable research data
Foundation models	Foundation models	Large pretrained models adaptable to multiple downstream tasks
GAMER	Reporting guideline for the use of generative artificial intelligence tools in medical research	Reporting guideline for generative AI use in medical research
Generative AI	Generative artificial intelligence	AI systems capable of generating text, images, or other content
GPT	Generative pre‐trained transformer	Family of transformer‐based generative language models
Human‐in‐the‐loop	Human‐in‐the‐loop	Workflow requiring human oversight and verification of AI outputs
IVD	Intervertebral disc	Fibrocartilaginous structure between adjacent vertebral bodies
JSON	JavaScript object notation	Lightweight structured data exchange format
LabOP	Laboratory open protocol specification	Standard for machine‐readable laboratory protocols
LLM	Large language model	Transformer‐based model trained on large text corpora for language understanding and generation
MedMCQA	Medical multiple‐choice question answering	Benchmark dataset for medical question answering
Med‐PaLM	Medical pathways language model	Medical‐domain large language model developed for biomedical question answering
MicroLLM	MicroLLM	LLM + NER pipeline for structured microbiology information extraction
ML	Machine learning	Algorithms that learn patterns from data without explicit programming
MRI	Magnetic resonance imaging	Imaging modality based on magnetic fields and radiofrequency signals
NER	Named entity recognition	NLP task identifying and classifying entities within text
NLP	Natural language processing	Computational processing and analysis of human language
OBI	Ontology for biomedical investigations	Controlled vocabulary describing biomedical investigations and experiments
Ontology	Ontology	Formal structured vocabulary defining concepts and relationships
ORS	Orthopedic Research Society	Scientific society relevant to musculoskeletal and spine research
PICO	Population–intervention–comparator–outcome	Framework for structuring research questions
PREPARE	Planning research and experimental procedures on animals: recommendations for excellence	Planning guideline for animal research and experimental procedures
PRISMA‐ScR	Preferred reporting items for systematic reviews and meta‐analyses extension for scoping reviews	Reporting framework for scoping reviews
PROM	Patient‐reported outcome measure	Standardized measure capturing health status, symptoms, or function directly from patients
Prompt engineering	Prompt engineering	Systematic design of model inputs to optimize outputs
ProtoCode	ProtoCode	LLM approach converting publications into machine‐readable protocols
PubMedQA	PubMed question answering dataset	Biomedical question answering benchmark derived from PubMed
QA	Question answering	Task in which a system generates or selects answers to questions
RAG	Retrieval‐augmented generation	Architecture combining document retrieval with LLM text generation
RF	Random forest	Ensemble machine‐learning method based on multiple decision trees
scGPT	Single‐cell generative pre‐trained transformer	Foundation model for single‐cell omics analysis
SCI	Spinal cord injury	Injury to the spinal cord with potential neurological consequences
SciScore	SciScore	Automated tool assessing rigor and reporting quality in biomedical studies
Semantic indexing	Semantic indexing	Meaning‐based indexing and retrieval of scientific text
SOP	Standard operating procedure	Formalized written protocol for reproducible procedures
SVM	Support vector machine	Supervised machine‐learning method for classification or regression
SYRCLE	Systematic review centre for laboratory animal experimentation	Risk‐of‐bias framework for animal studies
Transformer	Transformer	Attention‐based neural network architecture underlying modern language models
XGBoost	Extreme gradient boosting	Gradient‐boosted decision‐tree algorithm widely used for predictive modeling

### 2.5 Figure Preparation

Figures were primarily assembled in Microsoft PowerPoint, with selected scientific icons generated using ChatGPT‐5.2 (Pro, image mode) and subsequently curated and integrated by the authors.

### 2.6 Use of Large Language Models

ChatGPT‐5.2 (Pro version) was used in a supportive capacity for text structuring, language refinement, and formatting. Evidence identification, study selection, data interpretation, and all scientific conclusions were performed by the authors. The final manuscript was critically reviewed and approved in full by all authors, who assume responsibility for its accuracy and integrity.

## 3 Results

**FIGURE 1:**
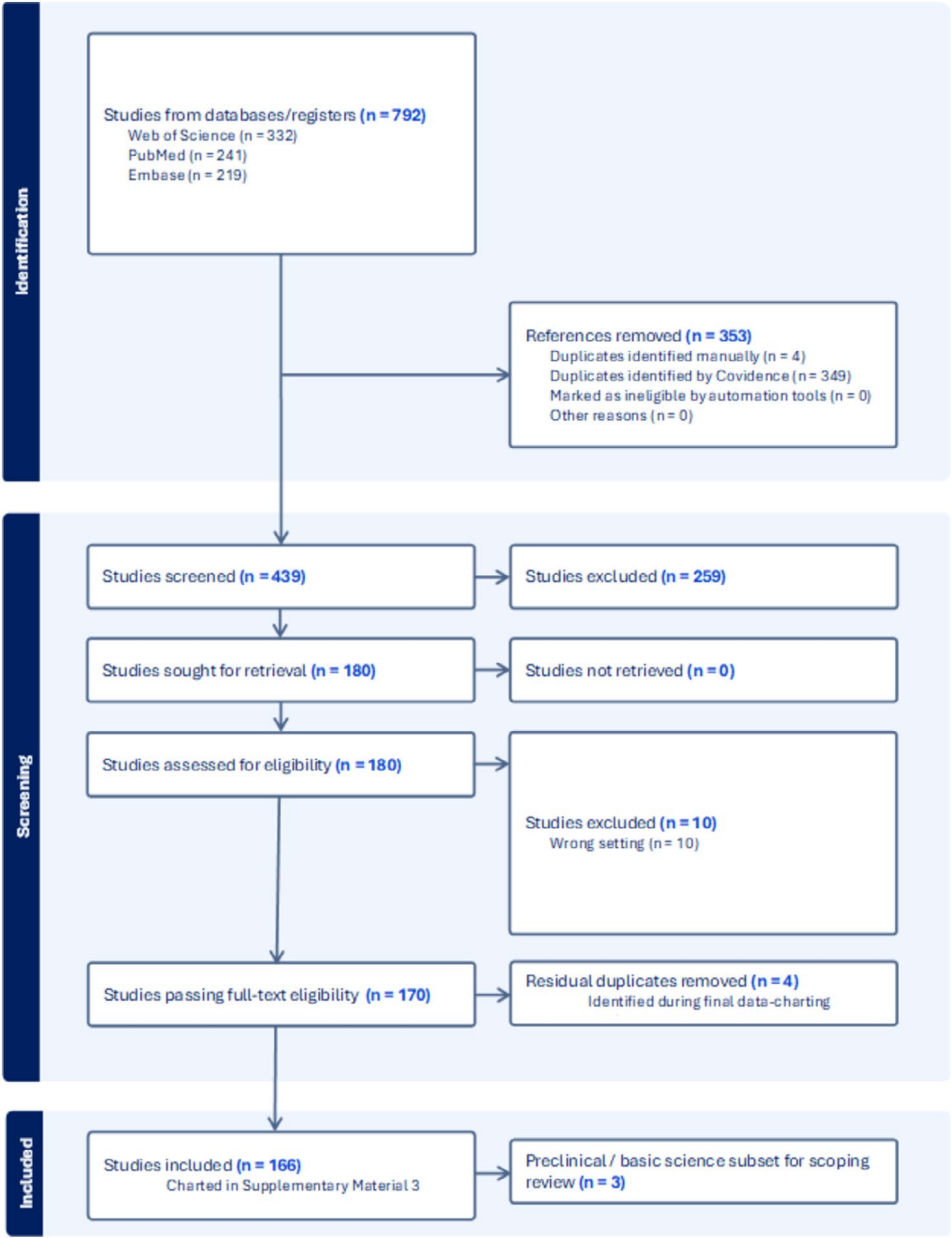
PRISMA flow diagram of study selection.

A total of 792 records were identified from database searches, including Web of Science (*n* = 332), PubMed (*n* = 241), and Embase (*n* = 219). After removal of 353 duplicates, 439 records remained for title and abstract screening. During screening, 259 records were excluded, leaving 180 reports sought for retrieval. Records excluded during title and abstract screening were outside the spine‐related scope, did not evaluate an LLM/chatbot/AI component, represented an ineligible publication type, or did not meet the predefined language criteria. All reports were retrieved and assessed for full‐text eligibility. At the full‐text stage, 10 reports were excluded because they concerned an ineligible setting. During final verification of the included‐study list, four additional duplicate records were identified and removed, resulting in 166 unique studies included in the final scoping review dataset (Figure 1). Publication activity increased markedly over time, with 1 study published in 2021, 1 in 2022, 13 in 2023, 48 in 2024, 94 in 2025, and 9 in 2026, the latter reflecting the partial search year ending in January 2026. The included literature was dominated by LLM/chatbot conversational assessment and patient‐reported outcome measure applications (82/166; 49.4%), followed by patient education and information quality evaluations (53/166; 31.9%) and other LLM/AI applications (19/166; 11.4%). Smaller categories included preclinical/basic science applications (3/166; 1.8%), imaging‐focused AI/ML applications (3/166; 1.8%), predictive modeling and decision support (3/166; 1.8%), surgical documentation/planning (1/166; 0.6%), patient Q&A/clinical vignette evaluation (1/166; 0.6%), and informed consent/shared decision‐making (1/166; 0.6%). Only three studies (3/166; 1.8%) addressed preclinical/basic science applications, underscoring the marked scarcity of translational laboratory‐focused AI work in the current spine literature. Notably, not all included studies were necessarily cited in the expert narrative synthesis; rather, the mapped corpus served to characterize the overall evidence landscape of LLM applications in spine care and research.

### 3.1 Preclinical and Basic Science Applications: Detailed Analysis

A central finding of this scoping review is the scarcity of preclinical AI work itself, which demonstrates that preclinical spine research remains virtually untouched by LLM technology and the broader family of advanced AI systems, despite rapidly expanding clinical applications. Importantly, while our search was designed to capture LLM‐specific applications, the three preclinical studies identified employed related but distinct AI/ML architectures (supervised and unsupervised machine learning, including deep neural networks) rather than generative LLMs in the narrow sense. This observation further underscores that even within the broader AI landscape, preclinical spine applications remain remarkably sparse. We therefore use the term “LLMs” in this manuscript to refer to the wider ecosystem of transformer‐based and advanced AI systems that process, structure, and reason over biomedical text and data.

Melgoza et al. developed a standardized histopathology scoring system for intervertebral disc (IVD) degeneration in the mouse model using machine learning algorithms, representing an ORS Spine Section initiative [5]. The authors introduced a comprehensive 14‐category histopathological scoring scheme and applied both supervised and unsupervised ML algorithms, including artificial neural networks, to quantitate degenerative changes in murine IVDs. The system achieved high sensitivity and specificity, demonstrating that AI‐based evaluation can reduce inter‐observer variability and increase the throughput and reproducibility of standardized scoring. This work established a critical proof‐of‐concept for AI‐assisted phenotyping in preclinical spine models and directly informs the consensus‐based grading frameworks discussed later in this review.

Alini et al. provided a comprehensive update on animal models of IVD degeneration and low back pain, explicitly exploring the potential of deep learning to improve research analysis and development of prospective therapeutics [6]. This review highlighted several preclinical AI applications: (i) AI‐assisted histopathological scoring using both murine (14‐category) and ovine (27‐point, 6‐category) grading schemes that could be enhanced by ML‐based evaluation to obviate inter‐observer variation; (ii) AI‐facilitated facial recognition pain assessment in animal IVD models, offering the possibility of correlating pain‐alleviating properties of therapeutic compounds with IVD regeneration; and (iii) deep learning‐improved clinical spinal imaging with direct translational relevance for preclinical model assessment. The authors concluded that AI may represent a paradigm shift in resolving the complexity of IVD degeneration and that implementation of AI methodology would improve the sensitivity and accuracy of preclinical evaluations.

Maity et al. synthesized post‐2019 advances in AI across key spine research application domains, including imaging analysis, predictive modeling, qualitative phenotyping, and emerging hybrid and language‐based frameworks [2]. While primarily clinically oriented, the authors discussed natural language processing (NLP) and LLM paradigms with direct translational relevance for preclinical workflows, including automated extraction of structured data from unstructured clinical text (applicable to laboratory documentation), hybrid modeling combining imaging with biomechanical data (relevant to preclinical fusion and biomechanical studies), and unsupervised phenotyping approaches that could be adapted for preclinical model characterization. The authors emphasized that despite impressive internal performance metrics, persistent challenges in generalizability, interpretability, and context‐appropriate validation continue to constrain clinical deployment—challenges equally relevant to preclinical AI implementation.

Collectively, these three studies demonstrate that AI/ML applications in preclinical spine research are at an early but promising stage, with the strongest evidence base in histopathological scoring automation and imaging analysis. Notably, none of the three studies employed LLMs in the generative transformer sense (e.g., GPT‐4, Claude, Gemini); rather, they utilized classical ML and deep learning architectures. This gap between the emerging LLM capabilities demonstrated in clinical spine applications (e.g., patient education, conversational assessment, structured data extraction) and the absence of LLM‐specific preclinical applications represents the central opportunity addressed by the expert perspective that follows.

## 4 Expert Perspective: LLM Integration Opportunities Across the Preclinical Spine Research Lifecycle

The following sections develop a structured expert perspective on where and how LLMs can be most productively integrated across the preclinical spine research lifecycle. Because peer‐reviewed empirical evidence specifically addressing LLM applications in preclinical spine research is currently limited to the three studies identified above, these sections draw on transferable principles from adjacent biomedical domains. They are presented as a forward‐looking framework rather than as scoping‐review results.

### 4.1 Large Language Models as “Co‐Scientists” in Preclinical Spine Research—Enabling the Next Layer of Evidence‐Aware Experimental Design

Preclinical spine research produces large volumes of often unstructured experimental knowledge and faces a persistent translational barrier. Experimental models of intervertebral disc degeneration and regeneration, spinal fusion, spinal cord injury and others remain heterogeneous, incompletely documented, and often poorly interoperable. This limits reproducibility, cross‐laboratory comparability, and secondary data use. Methodological heterogeneity and insufficient reporting have repeatedly been identified as major contributors to irreproducibility in musculoskeletal and spine research [7, 8]. These challenges mirror the broader reproducibility crisis in preclinical biomedical science, where many landmark findings cannot be consistently replicated across laboratories [9]. In spine research specifically, the translational gap has been repeatedly highlighted by the limited clinical translation of intervertebral disc regeneration strategies, the variability of spinal fusion outcomes across animal models, and the absence of harmonized endpoints linking preclinical findings to human disease [10, 11]. In practice, variability in “small” experimental details, such needle gauge/depth and level in disc puncture models, implant positioning and graft choice in fusion models, or perioperative care and analgesia or imaging acquisition as a broad theme in animal testing, can drive large differences in effect size and interpretability, yet these factors are often captured only narratively and inconsistently across publications and laboratories. High variability exists in in vitro and ex vivo paradigms (e.g., differences in cell source/donor metadata, passage number, scaffold composition, loading regimens), which can similarly drive divergent outcomes while remaining inconsistently documented across studies [12].

LLMs have clear potential to address these challenges with emerging consensus that the most defensible role of LLMs in biomedical research lies in constrained, human‐supervised applications embedded in validated workflows rather than autonomous inference [13, 14]. Accordingly, we emphasize use cases in which LLMs function as “workflow instruments” for evidence‐grounded synthesis, structured data extraction, completeness checking, protocol formalization, and documentation support, rather than as autonomous scientific decision‐makers. In this chapter, we use the term LLMs to refer to general‐purpose or domain‐adapted transformer models that generate or extract text (and, increasingly, can interface with tools and multimodal inputs), and we distinguish these from other AI systems commonly used in preclinical spine research (e.g., computer vision for image quantification or deep learning for omics).

To our knowledge, no prior review has specifically mapped the potential role of LLMs across the full preclinical spine research lifecycle. Given that literature specifically addressing LLMs in preclinical spine research is still scarce, this chapter necessarily draws on broader applications in biomedical science to outline transferable principles and emerging frameworks that may guide future spine‐specific adaptation and implementation. Accordingly, where possible, we highlight spine‐relevant entry points for LLMs as we believe that these applications can improve reproducibility without altering scientific interpretation.

### 4.2 From Evidence Synthesis and Hypothesis Generation to Experiment Planning

**FIGURE 2:**
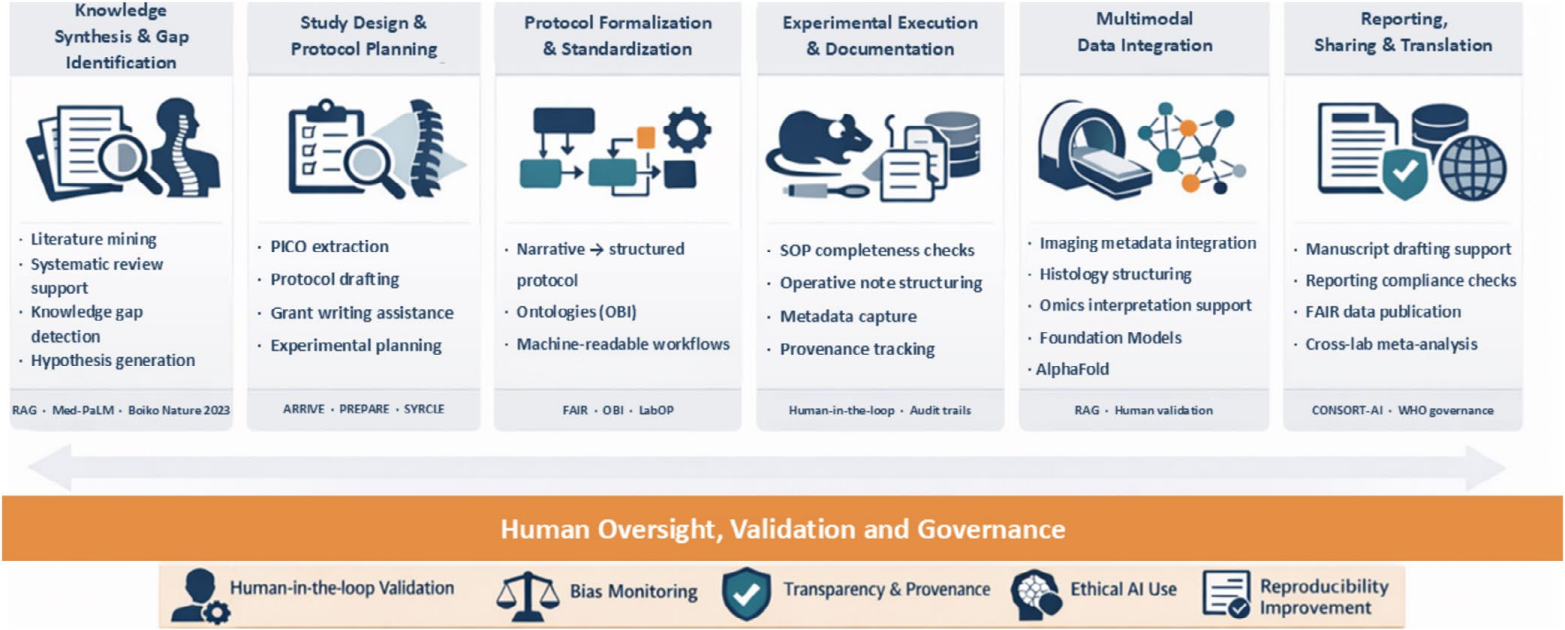
LLMs as co‐scientists: Augmented workflow for preclinical spine research. Schematic overview of how large language models (LLMs) could support the full preclinical spine research pipeline while remaining embedded in human‐supervised workflows. The horizontal pipeline illustrates six stages: (1) knowledge synthesis and gap identification; (2) study design and protocol planning; (3) protocol formalization and standardization; (4) experimental execution and documentation; (5) multimodal data integration; (6) reporting, sharing, and translation. ARRIVE, animal research: reporting of in vivo experiments guideline; CONSORT‐AI, consolidated standards of reporting trials–artificial intelligence; FAIR, findable–accessible–interoperable–reusable data principles; LabOP, laboratory open protocol specification; LLM, large language model; MRI, magnetic resonance imaging; OBI, ontology for biomedical investigations; PICO, population–intervention–comparator–outcome framework; PREPARE, planning research and experimental procedures on animals: recommendations for excellence; RAG, retrieval‐augmented generation; SOP, standard operating procedure; SYRCLE, systematic review centre for laboratory animal experimentation risk‐of‐bias tool; WHO, world health organization; μCT, micro‐computed tomography.

LLMs are increasingly being integrated across the entire preclinical research lifecycle (Figure 2) to support literature synthesis, conceptualization of research questions, development of methods, and research communication. In the context of literature review, domain‐specific LLM pipelines have been shown to be a promising adjunct to streamline systematic reviews and knowledge synthesis by automating retrieval and summarization while maintaining high fidelity to the evidence base, suggesting a scalable alternative to traditional manual review workflows [15, 16]. Beyond narrative summarization, LLM‐assisted review pipelines can support earlier systematic‐review steps that are typically rate limiting, including title/abstract screening assistance, extraction of structured study characteristics across in vitro, ex vivo, and in vivo designs (e.g., model system, species/strain/sex/age where applicable, cell source and passage, induction method, intervention, endpoints, and timepoints), and pre‐population of study‐quality or bias‐assessment fields using design‐appropriate tools (e.g., Systematic Review Centre for Laboratory animal Experimentation (SYRCLE) domains for animal studies [17]). Kurland et al. demonstrated that coupling GPT‐4 with an automated, bibliometrics‐powered literature search enabled accurate summarization and citation of key findings across common spinal pathologies, achieving 97.5% citation accuracy [18]. Beyond summarization, LLMs demonstrate the ability to generate novel and contextually grounded research hypotheses, pointing toward directions that might be overlooked due to human cognitive limits [13, 19, 20]. To make LLM‐generated hypotheses usable in preclinical spine research, each proposed mechanism can be accompanied by a measurable readout (e.g., imaging/biomechanics/histology/omics endpoint), the assumptions behind it, competing mechanisms, and the specific controls needed to discriminate them, with citations provided from retrieved sources. In protocol and objective generation, clinical and biomedical research reviews highlight that LLMs can assist in extracting structured trial elements and scientific rationale, such as PICO components, and support protocol design when augmented with retrieval and verification frameworks [21]. In preclinical spine contexts, analogous structured elements can include model‐defining parameters across cell/organ culture and animal studies, such as cell source/donor and passage, culture format and conditions (e.g., 3D matrix/scaffold, oxygen tension, mechanical loading), induction parameters (e.g., cytokine/enzymatic injury or disc puncture/fusion/SCI parameters), imaging acquisition/reconstruction settings (e.g., microscopy/μCT/MRI), and pre‐specified inclusion/exclusion criteria; all of these are variables that often drive cross‐study heterogeneity but are commonly inconsistently specified. Peer‐reviewed studies further show that LLM‐augmented research pipelines can meaningfully accelerate scientific discovery workflows when embedded in retrieval‐augmented generation (RAG) architectures that combine language modeling with curated evidence retrieval [22, 23]. For instance, Boiko et al. presented the development and capabilities of an AI system driven by GPT‐4, “Coscientist” that is capable of autonomously designing, planning and performing complex experiments by incorporating LLMs empowered by tools such as internet and documentation search, code execution and experimental automation [23]. While such systems illustrate the upper bound of autonomy, their most immediately transferable value to preclinical spine research may lie in tool‐augmented, constrained workflows (e.g., literature‐to‐protocol drafting, checklist‐driven completeness checking, and parameter standardization) rather than unsupervised experimental decision‐making. When grounded in retrieval‐augmented architectures and human validation loops, such systems can reduce drafting burden while preserving factual accuracy and transparency [22]. However, because errors can propagate rapidly in automated pipelines, LLM‐assisted outputs should pass through predefined human review checkpoints (parameter verification, citation checks, and investigator sign‐off) to preserve traceability and accountability. Together, these findings suggest that LLMs can support the conceptual phase of the research lifecycle while remaining embedded in human‐validated workflows.

### 4.3 Anchoring LLMs to Rigor and Reporting Frameworks

Importantly, meaningful LLM integration requires anchoring their use to established methodological standards that define rigor, transparency, and reproducibility. ARRIVE (Animal Research: Reporting of In Vivo Experiments) 2.0 defines minimum reporting requirements for animal studies and emphasizes that, without the “Essential 10,” the reliability of findings cannot be adequately judged [24]. PREPARE (Planning Research and Experimental Procedures on Animals: Recommendations for Excellence) complements ARRIVE by focusing on the practical and quality‐assurance dimensions of animal research planning [25]. For non‐animal preclinical work, analogous rigor expectations include transparent reporting of sample provenance (donor/source, inclusion criteria), replicates (biological vs. technical), randomization/blinding where applicable, and full specification of culture and perturbation conditions. However, these elements are often incompletely captured despite being critical determinants of reproducibility.

Bias and rigor frameworks further clarify what “good documentation” must capture. The SYRCLE risk‐of‐bias tool provides an established structure for evaluating common bias domains in animal studies [17]. In parallel, automated rigor scoring systems such as SciScore and the Rigor and Transparency Index demonstrate that key methodological criteria, including randomization, blinding and resource identification can be detected computationally and summarized as quality metrics [26]. These frameworks highlight a practical opportunity for LLMs: not to “judge” study quality, but to extract the relevant rigor fields, flag missing or ambiguous reporting, and produce a structured checklist for human confirmation. To make such checks actionable in spine research, community‐agreed minimum reporting checklists can complement general guidelines by capturing model‐specific details that routinely drive heterogeneity. Examples include disc degeneration models (e.g., induction method and severity grading; puncture gauge/depth/level or cytokine/enzymatic dose and duration; loading regimen), spinal fusion paradigms (level, fixation approach, graft type, endpoint definition and μCT acquisition/reconstruction thresholds), and SCI models (injury device and impact parameters, post‐operative care/rehabilitation, and pre‐specified exclusion criteria). LLM‐assisted templates and checklists can help standardize these details at the protocol and manuscript stages and reduce omission errors, while final responsibility remains with investigators.

Although developed for clinical trials, the CONSORT‐AI (Consolidated Standards of Reporting Trials—Artificial Intelligence) extension offers consensus‐based guidance on transparent reporting of AI systems, including their inputs and outputs, human–AI interaction, implementation context, and error analysis that could be adapted to preclinical spine research to strengthen rigor, interpretability, and reproducibility of AI‐augmented experimental workflows [27]. In parallel, the GAMER (Reporting guideline for the use of Generative Artificial intelligence tools in MEdical Research) statement provides the first universal, standardized reporting checklist for the use of generative AI tools across all types of medical research. Developed through an international Delphi consensus of 51 experts from 26 countries, GAMER comprises nine items covering tool specifications, prompting techniques, content verification, data privacy, and impact on conclusions, and is applicable to any study phase from design through manuscript preparation. Both extensions are catalogued under the EQUATOR Network registry of reporting guidelines [28], which we recommend as the central reference repository for AI‐related reporting extensions as they emerge. Adopting CONSORT‐AI and GAMER alongside domain‐specific frameworks such as ARRIVE and PREPARE would ensure that both the experimental rigor and the AI‐assisted components of preclinical spine research are transparent and reproducibly reported.

### 4.4 LLMs And the 3Rs: Reducing Animal Use While Improving Rigor and Translational Alignment

The 3Rs—Replacement, Reduction, and Refinement—provide a widely adopted ethical and scientific framework for animal research and are embedded in national and international policies and regulations [29]. This framing is particularly prominent in preclinical spine research, where animal studies not only commonly show substantial between‐study inconsistency but also variability between animals within the same cohort (e.g., related to disease induction efficacy), contributing to inconsistent effect sizes and limited reproducibility [8, 30]. As spine research progresses toward larger animal models as a late preclinical step, the financial and ethical costs of irreproducible or underpowered studies increase substantially, strengthening the need for workflows that maximize reproducibility and information yield per animal and reduce avoidable repetition.

**FIGURE 3:**
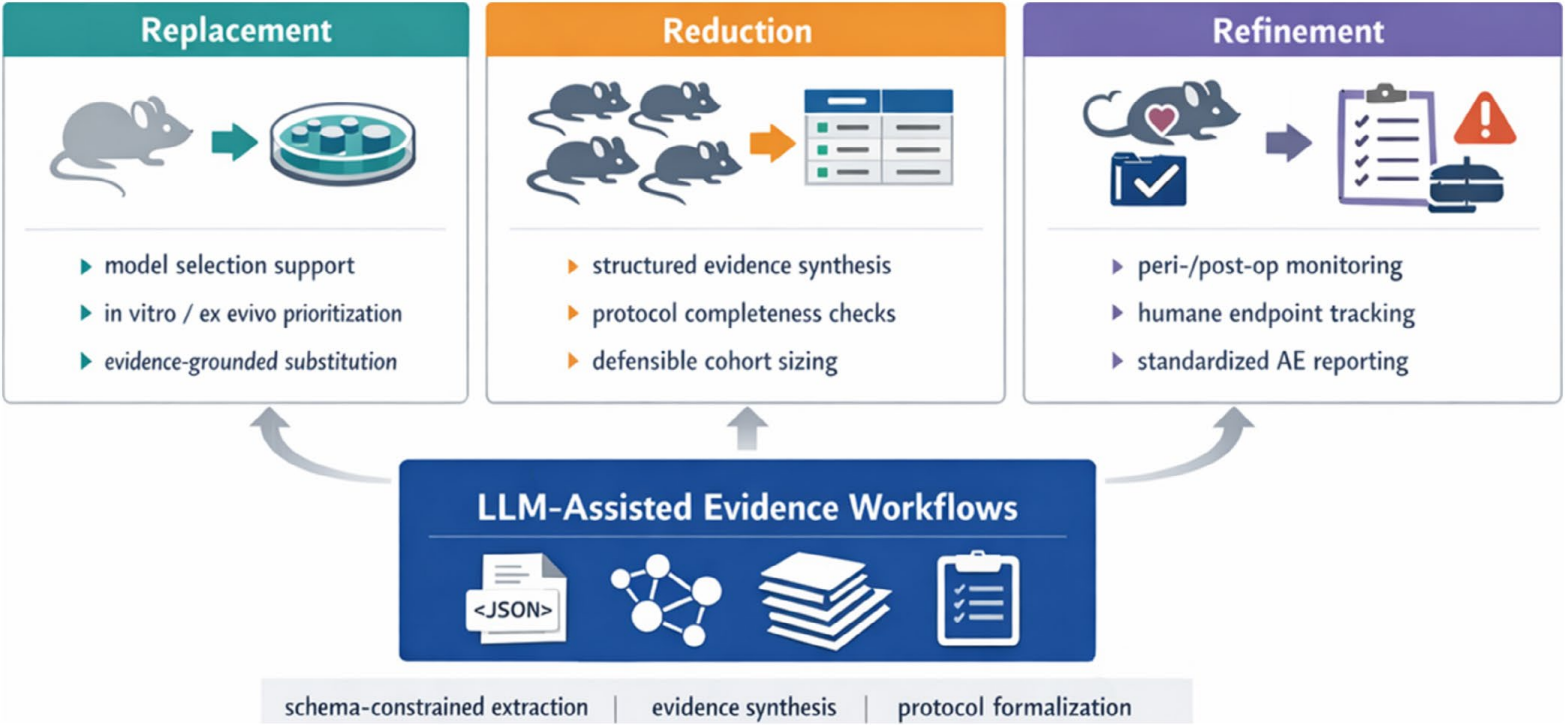
LLM‐enabled optimization of the 3Rs in preclinical spine research. Schematic overview of how LLM–assisted evidence workflows support replacement, reduction, and refinement within human‐supervised preclinical spine research. The central LLM layer enables schema‐constrained extraction, structured evidence synthesis, and protocol formalization, which inform (i) model selection and in vitro/ex vivo prioritization (Replacement), (ii) evidence‐based cohort planning and reporting completeness (Reduction), and (iii) peri−/post‐operative monitoring and adverse event (AE) report standardization (Refinement).

LLM‐enabled, evidence‐grounded workflows can support *Reduction* without weakening scientific rigor in at least three practical ways (Figure 3). First, structured evidence synthesis (e.g., LLM‐assisted screening, extraction, and evidence table generation) can help identify what has already been tested, where results are consistent or conflicting, and which gaps are truly unresolved, thereby reducing redundant experiments and improving prioritization. Second, protocol drafting and completeness checking against established planning and reporting guidelines (e.g., ARRIVE 2.0 and PREPARE [31]) can reduce preventable study failures driven by missing controls, ambiguous endpoints, or under‐specified methods—issues that can lead to unusable datasets and repeated animal cohorts. Third, by enabling more consistent reuse of prior preclinical data (including extraction of variance estimates and effect sizes from comparable models), LLM‐assisted pipelines can support more defensible cohort‐size planning and reduce both underpowered and unnecessarily large studies.

LLMs can also facilitate *Refinement* by strengthening documentation and monitoring practices that directly impact animal welfare and data integrity. For example, systematic capture of perioperative and post‐operative information (analgesia, antibiotics, monitoring schedules, and predefined humane endpoints), coupled with automated completeness checks, can reduce omissions and variability in animal care documentation. The PREPARE guidelines [32] explicitly emphasize planning‐stage practices that improve both animal welfare and study quality. In addition, structured logging enables early anomaly detection (e.g., rapid weight loss, unexpected adverse events, deviations from post‐op regimens), prompting timely review and humane interventions. Finally, standardized adverse event reporting, supported by LLM‐assisted templating and harmonized terminology, can reduce underreporting and accelerate cross‐laboratory learning, enabling refinement of procedures over time.

LLMs may also support *Replacement* (or partial replacement) by helping researchers select physiologically relevant in vitro or ex vivo systems and support decision making when these approaches may effectively substitute whole‐animal experiments. In practice, the barrier is often not the absence of alternative models but uncertainty about which model configurations (e.g., 3D culture systems, explant/organ culture, co‐culture designs, and mechanical stimulation regimens) best match a given biological question [33, 34]. Evidence‐grounded synthesis can support transparent “model selection” decisions, matching the scientific question to the lowest‐burden experimental system likely to yield valid inference while documenting the rationale for when escalation to in vivo work is truly necessary.

Critically, 3Rs‐oriented LLM use must be implemented to strengthen animal welfare and scientific rigor, not to justify weaker standards. This requires clear human accountability (defined sign‐off points) and traceable documentation of what the system did and did not do. These governance elements are consistent with the broader consensus that LLMs are most defensible in constrained, human‐supervised roles embedded in validated workflows.

### 4.5 FAIR Data, Ontologies, and Machine‐Readable Protocols

A persistent real‐world bottleneck in spine research is that critical methodological details remain buried in narrative publications and laboratory documents, limiting reproducibility, cross‐study comparability, and secondary data use, an area where LLM‐assisted structuring may offer tangible leverage. Ontology‐based information extraction has been demonstrated for preclinical SCI treatment publications using structured ontologies and entity linking [35] and machine‐assisted evidence synthesis tools have shown feasibility for accelerating structured reviews [36]. The ability to reason over scientific text has improved markedly with instruction‐tuning and chain‐of‐thought prompting, which enable models to perform multi‐step reasoning tasks [37]. This capability builds on decades of work in biomedical question answering and semantic indexing, including BioASQ [38], and medical question‐answering benchmarks such as PubMedQA and MedMCQA [39, 40]. Recent generative biomedical LLMs such as BioGPT further extend these capabilities to text generation and literature mining [41]. These developments allow LLMs to move beyond retrieval toward synthesis, comparison, and explanation of scientific findings.

Because data structuring is as important as reporting, LLM workflows should be aligned with Findable–Accessible–Interoperable–Reusable (FAIR) principles so that outputs become findable, accessible, interoperable, and reusable [42]. Operationally, this typically requires persistent identifiers (e.g., for samples, datasets, protocols, and reagents) and standardized metadata packaging so that records remain linkable and reusable across sites. Ontologies enable semantic interoperability at scale. Ontology for Biomedical Investigations (OBI) was developed specifically to describe biomedical investigations across planning, execution, and reporting using controlled vocabulary [43]. On the protocol layer, formal representations such as LabOP demonstrate how protocols and execution records can be modeled as machine‐readable workflows, which is highly relevant for preclinical surgery‐driven paradigms where deviations, parameter choices, and execution context often determine outcome validity [44]. Within this framework, LLMs' role can be envisioned to translate narrative protocol text into structured representations, check completeness against standards, and document human decisions and deviations. Such workflows represent a shift from narrative documentation toward computable experimental records. “ProtoCode”, for example, demonstrates that fine‐tuned LLMs can extract protocol elements from publications and translate them into machine‐readable representations with performance that depends on information density, illustrating the general paradigm “text to structured protocol object” as a reproducibility lever rather than a domain‐specific novelty [45]. For preclinical spine Standard Operating Protocols (SOPs), this implies a clear target of converting narrative text from SOPs, lab notes, and manuscripts into detailed, parameter‐explicit steps, which can then be validated against ARRIVE/PREPARE and spine‐specific mandatory fields. This is precisely the point at which LLMs can improve reproducibility without interfering with science. In practice, these elements transform LLMs from generative “writers” into standardized documentation instruments. They can make implicit expectations explicit, reduce omission errors, and harmonize terminology, while scientific decisions remain with investigators. This is particularly relevant for emerging international spine research consortia and multi‐center preclinical studies.

### 4.6 From Narrative Text to Structured Experimental Data and Human‐In‐The‐Loop Workflows

Strong gains are expected where preclinical workflows generate narrative text that is otherwise difficult to reuse. Converting free‐text documentation into predefined, structured data fields through schema‐constrained LLM extraction would enable automated completeness checks, cross‐study comparability, and meta‐analytic reuse [46]. This motivates schema‐first hybrid pipelines, in which predefined data models and controlled value sets constrain LLM extraction into machine‐readable outputs for example, JavaScript Object Notation (JSON) that can be automatically validated for completeness and plausibility before human sign‐off, thereby reducing ambiguity and limiting ungrounded generation [47]. In practice, these pipelines can include rule‐based and ontology‐informed validators (e.g., required fields, allowable ranges, unit normalization, and controlled vocabularies) that automatically flag missing or implausible values for review. Lu et al. developed “MicroLLM”, a structured information extraction tool that combines fine‐tuned LLMs with BERT‐based named entity recognition to extract complex, multi‐entity microbial phenotypic data from unstructured microbiology texts [48]. The system converts narrative content into structured JSON objects, reducing reliance on manual curation and enabling scalable construction of structured microbial knowledge bases for large‐scale bioinformatic analysis [48]. Importantly, the broader informatics and human‐centered AI literature stresses that such systems must include embedded validation, provenance, and human oversight to remain auditable and trustworthy, making review and verification integral pipeline components rather than optional post hoc steps [49, 50]. This design shifts LLMs from open‐ended generators toward structured data‐entry assistants that accelerate curation while preserving traceability and accountability.

### 4.7 Integration With Omics and Molecular Discovery

Omics approaches are increasingly central to preclinical spine research, including transcriptomic profiling of intervertebral disc degeneration, host–pathogen interaction studies in spinal infection and osteomyelitis, and molecular pathway analyses of osteogenesis and angiogenesis during spinal fusion. These rapidly growing datasets highlight the need for AI‐assisted interpretation frameworks capable of integrating high‐dimensional molecular data with experimental context [51, 52, 53]. In this setting, the value of LLM‐centered workflows is often greatest at the interface between molecular results and experimental metadata, that is, when models can help organize, standardize, and narratively interpret outputs from established analysis pipelines in a way that remains traceable to the underlying data. In structural biology, deep learning has already transformed protein science. AlphaFold demonstrated near‐experimental accuracy in protein structure prediction and enabled large‐scale structural annotation of proteomes [54]. Building on similar transformer principles, generative sequence models have successfully produced functional proteins with experimentally validated activity, illustrating that LLM systems can move from passive annotation toward hypothesis‐driven molecular design [55]. Although these advances are not spine‐specific, they illustrate how foundation models can accelerate target prioritization and molecular reagent design when paired with empirical validation. In parallel, multi‐omics integration frameworks increasingly rely on deep learning to harmonize transcriptomic, proteomic, and pathway‐level data, enabling cross‐modal inference that exceeds classical enrichment pipelines. LLMs show potential for assisting in clustering redundant gene sets, identifying upstream regulators across datasets, and translating high‐dimensional signatures into mechanistically coherent hypotheses [56, 57]. A practical and defensible role is to use LLMs to (a) summarize results from validated statistical workflows (e.g., differential expression, pathway enrichment, network inference), (b) map outputs to standardized gene set and pathway nomenclature, (c) flag inconsistencies or missing metadata, and (d) generate structured, evidence‐linked interpretation notes. Cui et al. introduced a foundation model for single‐cell biology, scGPT, based on a generative pretrained transformer across a repository of over 33 million cells [58]. They suggested that scGPT can be optimized to achieve superior performance across diverse downstream applications, including tasks such as cell type annotation, multi‐batch integration, multi‐omic integration, perturbation response prediction and gene network inference.

Given the documented tendency of LLMs to generate plausible but non‐verifiable statements in unconstrained settings [59, 60], their role in omics analysis should be limited to structured, evidence‐bound interpretation layers that operate downstream of validated statistical pipelines rather than replacing them. Collectively, these findings suggest that AI systems have the potential to augment the management of large omics databanks, pathway interpretation, and molecular reagent selection, thereby strengthening translational inference without displacing empirical validation.

### 4.8 Histopathology and Behavioral Phenotyping

Histopathology provides a particularly favorable entry point because the spine community has already developed consensus‐based scoring infrastructures. Standardized disc degeneration grading systems, including JOR Spine consensus frameworks, provide stable “ground‐truth” fields for synoptic reporting and term mapping [5]. In this setting, LLMs may be potentially best used to generate structured templates, map free text to defined scoring fields, harmonize nomenclature, and perform completeness checks. A practical extension is “synoptic‐style” reporting, in which scoring outputs are captured in standardized, machine‐readable forms (e.g., predefined fields for key histologic features), and narrative text in manuscripts is generated from these structured entries rather than serving as the primary record [61]. Alini et al. highlight that AI can reduce observer variability and increase throughput in experimental disc research, particularly through automated histopathology quantification, imaging analysis, and facial‐recognition–based pain assessment in animal models of intervertebral disc degeneration and low back pain [6]. In LLM‐assisted workflows, computer‐vision–derived measurements (e.g., region‐level quantification, segmentation metrics, or automated scores) can be automatically paired with the associated metadata (model details, timepoints, staining protocols, imaging settings) and formatted into consistent reporting tables and methods‐compliant text with explicit provenance. More broadly, the combination of standardized scoring frameworks with automated quantification and LLM‐enabled structured reporting provides a clear path to reduce observer variability while improving cross‐study comparability and data reuse.

## 5 Summary, Limitations and Perspectives

LLMs have the potential to reshape preclinical spine research by converting fragmented experimental workflows into more interoperable and evidence‐aware systems. In the near term, their most credible value lies in supporting protocol standardization, structured data extraction, and evidence‐linked hypothesis generation within human‐supervised pipelines. However, current evidence in preclinical spine research remains sparse and largely extrapolated from broader biomedical domains. Only three of the 166 included studies addressed preclinical applications, and none employed LLMs in the generative transformer sense, instead utilizing classical ML and deep learning architectures. This underscores both the novelty and the limitations of the present analysis. Several methodological limitations warrant acknowledgment. First, our search strategy intentionally included both LLM‐specific and broader AI/chatbot terms, as these are frequently used interchangeably in the spine literature. This means that the 166‐study corpus includes studies using non‐LLM architectures (e.g., rule‐based chatbots, classical NLP), and results should be interpreted accordingly. Second, the search was restricted to peer‐reviewed databases (PubMed, Embase, Web of Science); preprint servers (bioRxiv, medRxiv, arXiv) were not searched, which may have excluded emerging work in this rapidly evolving field. Third, the expert perspective sections necessarily draw on literature outside the scoped corpus, as the preclinical evidence base alone is insufficient to support actionable recommendations; this design is transparently disclosed and the structural separation between scoping results and expert synthesis aims to maintain interpretive clarity. Spine‐specific benchmarking and prospective validation studies are therefore urgently needed to assess accuracy, reliability, and real‐world impact across multicenter experimental settings. Future evaluations should focus on clearly defined tasks such as parameter extraction, checklist‐based completeness assessment, and evidence‐grounded synthesis using domain‐specific gold standards. Transparent governance, including audit trails, explicit handling of missing information, and predefined human verification checkpoints, will be essential to ensure responsible implementation. Rather than replacing investigators, LLMs are best positioned as adaptive research copilots that enhance reproducibility, reduce avoidable variability, and support more coordinated preclinical spine research ecosystems.

Beyond the experimental domain, LLMs may ultimately serve as semantic bridges across the spine research continuum by aligning phenotypes, operationalizing scores and classifications into computable units, and preserving nuance when translating narrative observations into structured variables. This translational interface, particularly between clinical documentation and preclinical model design, represents a major but as yet incompletely explored opportunity and is planned to be addressed in a dedicated companion analysis derived from this methodological framework.

## 6 Conclusion

LLMs are emerging as pragmatic interface technologies for preclinical spine science, with their greatest near‐term value arising from structured, human‐supervised integration rather than autonomous reasoning. When anchored to rigorous reporting frameworks and interoperable data standards, these systems can transform narrative experimental knowledge into computable evidence streams that strengthen reproducibility and translational readiness. The next critical step is prospective, spine‐specific validation embedded within transparent and well‐governed experimental workflows.

## Author Contributions

**Victor E. Staartjes:** investigation, formal analysis, writing – review and editing, writing – original draft, visualization, validation. **Stefan Motov:** writing – original draft, writing – review and editing, investigation, formal analysis, validation. **Julian Scherer:** investigation, formal analysis, writing – review and editing, writing – original draft, validation. **Karin Wuertz‐Kozak:** formal analysis, writing – review and editing, writing – original draft, investigation, supervision, validation, project administration. **Jonas Krueckel:** formal analysis, investigation, writing – original draft, writing – review and editing, data curation, validation. **Siegmund Lang:** conceptualization, methodology, data curation, supervision, project administration, formal analysis, validation, investigation, writing – original draft, writing – review and editing, visualization.

## Funding

No specific funding was received for the conduct of this scoping review. Funding sources of individual included studies were not systematically extracted, as the objective was to map application domains and evidence maturity rather than to perform comparative effectiveness synthesis.

## Conflicts of Interest

The authors declare no conflicts of interest.

## Supporting information


**Data S1:** Electronic search strategy. Complete search strings and field restrictions applied in PubMed (MEDLINE), Embase (Ovid), and Web of Science Core Collection, including the combination of large language model and conversational‐AI terms with spine‐specific terminology.

**Data S2:** PRISMA‐ScR checklist. Completed Preferred Reporting Items for Systematic Reviews and Meta‐Analyses extension for Scoping Reviews (PRISMA‐ScR) checklist, with justification provided for items not applicable to the present hybrid scoping‐review and expert‐perspective design.

**Data S3:** Study characteristics (data‐charting table). Full data‐charting table summarizing all 166 included studies, reporting first author, year, journal, AI/LLM system(s) used, AI application characterization, thematic domain, study design, and spine pathology focus.

## Data Availability

The data that support the findings of this study are available from the corresponding author upon reasonable request.
