# Supplementary material for: Large Language Models in Preclinical Spine Research: A Scoping Review and Expert Perspective on Evidence‐Aware Experimental Workflows
Source: JOR Spine. 2026 Jul 8;9(3):e70203. doi: 10.1002/jsp2.70203 (PMC13344227; doi:10.1002/jsp2.70203)
Supplement: Supplementary file 1 — Data S1: Electronic search strategy. Complete search strings and field restrictions applied in PubMed (MEDLINE), Embase (Ovid), and Web of Science Core Collection, including the combination of large language model and conversational‐AI terms with spine‐specific terminology. [file JSP2-9-e70203-s002.docx]

| **Supplementary Material 1. Search Strategy for the Focused Scoping Review** | |
| --- | --- |
| The final search was conducted on January 15, 2026. Searches covered publications from January 2020 to January 2026 and were limited to English and German language publications. Searches were restricted to title/abstract fields in PubMed and Embase and to topic fields in Web of Science. | |
| **Source** | **Syntax** |
| **PubMed** | (("large language model"[Title/Abstract] OR "large language models"[Title/Abstract] OR LLM[Title/Abstract]  OR LLMs[Title/Abstract] OR ChatGPT[Title/Abstract] OR "GPT-4"[Title/Abstract] OR Claude[Title/Abstract] OR Gemini[Title/Abstract] OR Llama[Title/Abstract] OR "Med-PaLM"[Title/Abstract] OR chatbot*[Title/Abstract] OR "conversational AI"[Title/Abstract]) AND (spine[Title/Abstract] OR spinal[Title/Abstract] OR vertebr*[Title/Abstract] OR "spinal surgery"[Title/Abstract] OR scoliosis[Title/Abstract] OR "disc herniation"[Title/Abstract] OR "spinal stenosis"[Title/Abstract] OR spondylodiscitis[Title/Abstract] OR "vertebral osteomyelitis"[Title/Abstract] OR "back pain"[Title/Abstract]) AND (surgery[Title/Abstract] OR surgical[Title/Abstract] OR patient*[Title/Abstract] OR clinical[Title/Abstract] OR cohort[Title/Abstract] OR registry[Title/Abstract] OR outcome*[Title/Abstract] OR perioperative[Title/Abstract] OR postoperative[Title/Abstract] OR preoperative[Title/Abstract] OR research[Title/Abstract] OR study[Title/Abstract] OR experimental[Title/Abstract] OR preclinical[Title/Abstract] OR translational[Title/Abstract] OR "in vitro"[Title/Abstract] OR "in vivo"[Title/Abstract] OR animal[Title/Abstract] OR biomechanic*[Title/Abstract] OR "finite element"[Title/Abstract] OR histolog*[Title/Abstract] OR radiomic*[Title/Abstract] OR "data extraction"[Title/Abstract] OR "scientific writing"[Title/Abstract] OR "literature review"[Title/Abstract])) |
| **EMBASE** | ("large language model*" OR LLM OR LLMs OR ChatGPT OR GPT-4 OR Claude OR Gemini OR Llama  OR "Med-PaLM" OR chatbot* OR "conversational AI").ti,ab. AND (spine OR spinal OR vertebr* OR "spinal surgery" OR scoliosis OR "disc herniation" OR "spinal stenosis" OR spondylodiscitis OR "vertebral osteomyelitis" OR "back pain").ti,ab. AND (surgery OR surgical OR patient* OR clinical OR cohort OR registry OR outcome* OR perioperative OR postoperative OR preoperative OR research OR study OR experimental OR preclinical OR translational OR "in vitro" OR "in vivo" OR animal OR biomechanic* OR "finite element" OR histolog* OR radiomic* OR "data extraction" OR "scientific writing" OR "literature review").ti,ab. |
| **Web of Science** | TS=(("large language model*" OR LLM OR LLMs OR ChatGPT OR "GPT-4" OR Claude  OR Gemini OR Llama OR "Med-PaLM" OR chatbot* OR "conversational AI") AND (spine OR spinal OR vertebr* OR "spinal surgery" OR scoliosis OR "disc herniation" OR "spinal stenosis" OR spondylodiscitis OR "vertebral osteomyelitis" OR "back pain") AND (surgery OR surgical OR patient* OR clinical OR cohort OR registry OR outcome* OR perioperative OR postoperative OR preoperative OR research OR study OR experimental OR preclinical OR translational OR "in vitro" OR "in vivo" OR animal OR biomechanic* OR "finite element" OR histolog* OR radiomic* OR "data extraction" OR "scientific writing" OR "literature review")) |
