# Supplementary material for: Large Language Models in Preclinical Spine Research: A Scoping Review and Expert Perspective on Evidence‐Aware Experimental Workflows
Source: JOR Spine. 2026 Jul 8;9(3):e70203. doi: 10.1002/jsp2.70203 (PMC13344227; doi:10.1002/jsp2.70203)
Supplement: Supplementary file 2 — Data S2: PRISMA‐ScR checklist. Completed Preferred Reporting Items for Systematic Reviews and Meta‐Analyses extension for Scoping Reviews (PRISMA‐ScR) checklist, with justification provided for items not applicable to the present hybrid scoping‐review and expert‐perspective design. [file JSP2-9-e70203-s001.docx]

**Supplementary Material 2: PRISMA-ScR Checklist**

**Manuscript title:** *Large Language Models in Preclinical Spine Research: A Scoping Review and Expert Perspective on Evidence-Aware Experimental Workflows*

| **Section** | **Item** | **PRISMA-ScR checklist item** | **Reported in section** | **Statement / location in manuscript** |
| --- | --- | --- | --- | --- |
| **TITLE** | **1** | Identify the report as a scoping review. | Title page | **Reported.** The title explicitly identifies the manuscript as “A Scoping Review and Expert Perspective.” |
| **ABSTRACT** | **2** | Provide a structured summary including background, objectives, eligibility criteria, sources of evidence, charting methods, results, and conclusions. | Abstract | **Reported.** The abstract includes Background, Methods, Results, and Conclusion. It describes the focused scoping search, databases, time frame, inclusion of LLM/AI/NLP applications, the 166 included studies, and the central preclinical evidence gap. |
| **INTRODUCTION** | **3** | Describe the rationale for the review in the context of what is already known. | Introduction | **Reported.** The manuscript explains the need for evidence-aware LLM workflows in preclinical spine research, emphasizing heterogeneous experimental reporting, semantic fragmentation, reproducibility barriers, and translational gaps. |
| **INTRODUCTION** | **4** | Provide an explicit statement of the questions and objectives being addressed with reference to key concepts, target population or sources of evidence. | Objective of this Scoping Review | **Reported.** The objective is stated as delineating and critically contextualizing emerging roles of LLMs within preclinical and experimental spine research, mapping high-yield use cases, evaluating evidence maturity, and identifying methodological constraints. |
| **METHODS** | **5** | Indicate whether a review protocol exists; state if and where it can be accessed. | Methods/ Structured Literature Search Strategy | No formal review protocol was registered for this focused scoping review and expert perspective. |
| **METHODS** | **6** | Specify characteristics of sources of evidence used as eligibility criteria and provide rationale. | Methods: Structured Literature Search Strategy | **Reported.** Eligibility criteria are stated: studies reporting development, evaluation, benchmarking, or implementation of language-model–based systems in spine surgery, spine patient care, or spine-related preclinical/translational research were included. Exclusion criteria included non-English/non-German publications, purely imaging-based AI without language-model components, robotics-only studies, and opinion pieces without methodological content. |
| **METHODS** | **7** | Describe all information sources and date of the most recent search. | Methods: Structured Literature Search Strategy | **Reported.** PubMed/MEDLINE, Embase/Ovid, and Web of Science Core Collection were searched. The final search date was January 15, 2026, covering January 2020 to January 2026. |
| **METHODS** | **8** | Present the full electronic search strategy for at least one database, including limits used, so that it could be repeated. | Methods and Supplementary Material 1 | **Reported.** The main text summarizes search terms and restrictions. Supplementary Material 1 includes the exact search strings for PubMed, Embase, and Web of Science. |
| **METHODS** | **9** | State the process for selecting sources of evidence, including screening and eligibility assessment. | Methods: Structured Literature Search Strategy | **Reported.** Records were imported into Covidence, deduplicated, screened by title/abstract independently by two reviewers, followed by duplicate full-text review. Disagreements were resolved by consensus. |
| **METHODS** | **10** | Describe the methods of charting data from included sources of evidence and processes for obtaining/confirming data. | Methods | **Reported.** Data were charted using a structured extraction table (Supplementary Material 3). Extracted fields included publication year, study design/type, application domain, AI/LLM system or method, and spine-related context. Data charting was reviewed by the author team for consistency. |
| **METHODS** | **11** | List and define all variables for which data were sought and any assumptions or simplifications made. | Methods and Supplementary Material 3 | **Reported.** Methods section (“Data Charting and Thematic Categorization”) and Supplementary Material 3. The following variables were charted for all 166 included studies: (1) first author and year, (2) title, (3) journal, (4) publication year, (5) DOI, (6) thematic category (9 predefined domains), (7) AI/LLM system(s) used (e.g., GPT-3.5, GPT-4, Gemini, Claude, DeepSeek, classical ML/DL), (8) AI application characterization, classified as generative LLM, unspecified LLM, conventional chatbot/non-LLM, classical/other AI/ML, hybrid LLM + ML/DL, AI general/unclear, or not specified/unclear, (9) study design, and (10) spine pathology focus. Charting was performed independently by two reviewers with discrepancies resolved by consensus. |
| **METHODS** | **12** | If done, provide rationale for conducting critical appraisal of included sources; describe methods and use in synthesis. | Methods | Formal critical appraisal was not performed because the review was designed to map evidence domains and identify gaps rather than to assess comparative intervention effectiveness. |
| **METHODS** | **13** | Describe methods of handling and summarizing the charted data. | Methods and Results | **Reported.** Charted data were summarized descriptively using frequencies and percentages for major application domains. Preclinical/basic science studies were then examined narratively in greater detail, and representative adjacent-domain studies were used to inform the expert-perspective synthesis. |
| **RESULTS** | **14** | Give numbers of sources screened, assessed for eligibility, and included, with reasons for exclusions at each stage, ideally using a flow diagram. | Results and Figure 1 | **Reported.** The manuscript reports 792 records identified, 353 duplicates removed before screening, 439 records screened, 259 excluded at title/abstract screening, 180 reports assessed for full-text eligibility, 10 reports excluded at full-text stage, and four residual duplicates removed during final data verification, resulting in 166 unique included studies. Figure 1 is described as the PRISMA flow diagram. |
| **RESULTS** | **15** | For each source of evidence, present characteristics for which data were charted and provide citations. | Results, Table 1, Supplementary Material 3 | **Reported.** The three preclinical/basic science studies are described in detail in the main manuscript and Table 1. The full characteristics of all 166 included studies are provided in Supplementary Material 3. |
| **RESULTS** | **16** | If done, present data on critical appraisal of included sources. | Results | **Not applicable.** Formal critical appraisal was not performed. |
| **RESULTS** | **17** | For each included source of evidence, present the relevant data that were charted and that relate to the review questions/objectives. | Results, Table 1, Supplementary Material 3 | **Reported.** The manuscript presents detailed data for the three preclinical/basic science studies and summarizes the broader 166-study corpus by application category. The complete charted evidence base are provided in Supplementary Material 3. |
| **RESULTS** | **18** | Summarize and/or present the charting results as they relate to the review questions and objectives. | Results | **Reported.** The manuscript summarizes the mapped corpus by publication year and thematic application domain. The Results describe the rapid temporal expansion of the literature and present the charted application domains, including conversational assessment and patient-reported outcome measure applications, patient education and information quality evaluations, other LLM/AI applications, preclinical/basic science applications, imaging-focused AI/ML applications, predictive modeling and decision support, surgical documentation/planning, patient Q&A/clinical vignette evaluation, and informed consent/shared decision-making. The manuscript identifies the scarcity of preclinical/basic science applications, and particularly the near-absence of LLM-specific preclinical spine applications, as the central finding. |
| **DISCUSSION** | **19** | Summarize the main results, including concepts, themes, types of evidence, and relevance to key groups. | Summary, Limitations and Perspectives; Expert Perspective sections | **Reported.** The manuscript summarizes the evidence gap, emphasizes the lack of generative LLM studies in preclinical spine research, and outlines relevant themes including evidence synthesis, protocol standardization, ARRIVE/PREPARE alignment, 3Rs optimization, FAIR data, ontologies, machine-readable protocols, structured data extraction, omics, histopathology, and behavioral phenotyping. |
| **DISCUSSION** | **20** | Discuss limitations of the scoping review process. | Summary, Limitations and Perspectives | **Reported.** The manuscript states that the search included both LLM-specific and broader AI/chatbot terms, that non-LLM architectures were included, that only peer-reviewed databases were searched, that preprint servers were not searched, and that expert-perspective sections necessarily draw on literature outside the scoped corpus. |
| **DISCUSSION** | **21** | Provide a general interpretation of results with respect to review questions/objectives and implications/next steps. | Conclusion; Summary, Limitations and Perspectives | **Reported.** The manuscript concludes that LLMs are best positioned as human-supervised workflow instruments for structured data extraction, protocol standardization, and evidence-linked synthesis, and calls for prospective, spine-specific validation and transparent governance. |
| **FUNDING** | **22** | Describe sources of funding for included sources of evidence and for the scoping review; describe role of funders. | Funding statement | No specific funding was received for the conduct of this scoping review. Funding sources of individual included studies were not systematically extracted, as the objective was to map application domains and evidence maturity rather than to perform comparative effectiveness synthesis. The authors declare that no funder had any role in study design, data interpretation, manuscript preparation, or the decision to submit. |
